# Ceramic-on-Ceramic Total Hip Arthroplasty: I Can Hear You

**DOI:** 10.1016/j.artd.2023.101203

**Published:** 2023-09-19

**Authors:** Zhida Shang, Michael Tanzer, Hamid Al Badi, Adam Hart

**Affiliations:** Jo Miller Orthopaedic Research Laboratory, Division of Orthopaedic Surgery, McGill University, Montreal, Quebec, Canada

**Keywords:** Squeaking, Ceramic, Arthroplasty, Hip, Bearing, Loudness, Noise

## Abstract

**Background:**

Squeaking is a known complication of ceramic-on-ceramic (CoC) total hip arthroplasty (THA), yet there is a lack of studies specifically quantifying its loudness. The aims of this study were: (1) to determine the incidence of squeaking in CoC THAs at long-term follow-up; (2) to identify risk factors; and (3) to quantify the loudness of the squeaking.

**Methods:**

A specifically designed prospective questionnaire was used to determine the prevalence, characteristics, and loudness of squeaking in 130 (110 patients) primary THAs with fourth-generation CoC bearings at a mean follow-up of 10.5 years. The loudness of the squeaking was determined by the decibel (dB) scale from the Centers for Disease Control and Prevention.

**Results:**

Overall, 28% of the CoC hips experienced squeaking. The mean onset was 5.7 years postoperatively, with 39% of the cases having their onset more than 5 years after their THA. Patients with a lower body mass index were more likely to report squeaking (*P* = .009). The mean loudness of the squeak was 35 dB (range, 10-70 dB) and was loud in 36% of the hips. Patients who developed squeaking early postoperatively had louder squeaking than those with a later onset (*P* = .007). The loudness of the squeaking sound progressed in 25% of the cases, and these hips had louder squeaking (*P* = .04).

**Conclusions:**

Squeaking after CoC THA is not uncommon, can be relatively loud, and increases over time. This needs to be considered in young patients that are candidates for CoC THAs.

## Introduction

Total hip arthroplasty (THA) has been widely used as an effective surgical intervention in managing many disabling hip conditions. Ceramic-on-ceramic (CoC) bearings are an attractive option for young patients due to their extremely low wear rates [[1], [2], [3]]. Squeaking is a known complication of CoC THA, with a reported incidence ranging from 2.4% to 53% [[4], [5], [6], [7], [8], [9]]. Squeaking occurs more frequently years after the THA [8], explaining the low incidences in studies with shorter follow-up [8,10]. Squeaking is not frequently associated with pain or functional impairment [5,11], and revision due to squeaking remains uncommon at 0.2% [4]. Microseparation, impingement, metal transfer, and edge loading causing stripe wear have been postulated as possible etiologies [12,13]. However, the exact cause has not been elucidated, with several studies reporting no significant associations [10,[14], [15], [16]], and others suggesting that the cause of squeaking is multifactorial [4,13,17]. Considering the low wear rates of contemporary highly cross-linked polyethylene bearings, the suitability of CoC bearings is more controversial and requires a thorough understanding of the long-term incidence, risk factors, and loudness of squeaking.

The characteristics and loudness of the squeaking can affect the psychosocial wellbeing of patients and may even necessitate revision surgery to eliminate the squeaking [18]. Despite its importance, the loudness of the squeaking and its progression remain unknown. Sound is measured in decibels (dB) and is used to measure the relative loudness of sounds. To date, studies have examined CoC squeaking as a qualitative variable, such as the perception of audible squeaking to others around the patient [11,13,19,20], limiting our understanding of the sound loudness caused by CoC THAs. To our knowledge, there are no studies quantifying the loudness of squeaking in CoC THAs and correlating the sound generated with it being audible to others. The aims of this study were: (1) to determine the incidence of squeaking in patients with CoC THA at long-term follow-up; (2) to examine risk factors for squeaking; and (3) to quantify the loudness of the squeaking.

## Material and methods

Institutional review board approval was obtained prior to the onset of the study. A retrospective review of our institution’s prospectively collected total joint registry was conducted to identify all patients undergoing primary THA with fourth-generation CoC bearings (Biolox delta, CeramTec AG; Plochingen, Germany) between July 2005 and September 2019, with a minimum follow-up of 2 years. The surgeon felt that a CoC bearing was indicated in these patients because they were younger and/or had a high physical activity level at a time when the long-term benefits of highly cross-linked polyethylene were still uncertain. Revision surgeries and other types of bearings were excluded. In total, 160 THAs (138 patients) met the criteria. Of these, 19 patients were lost to follow-up (21 hips), 2 patients were deceased (2 hips), and 7 hips (7 patients) with clicking or grinding were excluded, leaving 130 THAs (110 patients) for analysis. All 110 patients agreed to participate in the study.

All surgeries were done by a single surgeon (M.T.) through a minimally invasive posterior approach. A Pinnacle acetabular component with Gription porous coating (DePuy-Synthes, Johnson & Johnson, Warsaw, IN) was used in 126 hips; a Continuum cup (Zimmer-Biomet, Warsaw, IN) in 2 hips; a Trilogy cup (Zimmer-Biomet, Warsaw, IN) in 1 case; and a R3 cup in 1 hip (Smith-Nephew, Andover, MA). The femoral component was a Trilogy BPS stem (DePuy-Synthes, Johnson & Johnson, Warsaw, IN) in 65 hips, a S-ROM stem (DePuy-Synthes, Johnson & Johnson, Warsaw, IN) in 61 hips, a CLS stem (Zimmer-Biomet, Warsaw, IN) in 2 hips, a Summit stem (DePuy-Synthes, Johnson & Johnson, Warsaw, IN) in 1 hip, and an Anthology stem (Smith-Nephew, Andover, MA) in 1 hip. A Biolox delta ceramic head was used in all cases, and the femoral head size was 28 mm in 3 hips, 32 mm in 21 hips, and 36 mm in 106 hips.

The patients were followed for a mean of 10.5 years (range, 2.8-17 years). The mean age at the time of surgery was 50 years (range, 21-77 years). The patients were female in 53% of the cases and male in 47%. The THA involved the right hip in 57% of the cases and the left hip in 43% of the cases. The initial diagnosis was osteoarthritis in 73 cases, developmental dysplasia of the hip in 28 cases, osteonecrosis in 16 cases, inflammatory arthritis in 7 cases, septic arthritis in 2 cases, pigmented villonodular synovitis in 2 cases, idiopathic protrusio in 1 case, and traumatic in 1 case.

A specifically designed prospective telephone questionnaire developed by the researchers was administered to all patients to determine whether patients had squeaking from their CoC THA (Appendix). All noise other than squeaking was excluded from the study. The questionnaire evaluated the onset of squeaking and the activity that started it; its frequency; aggravating activities; if the patient avoided activities because of the squeaking; any associated pain or discomfort; the loudness of the squeaking; its progression in terms of frequency and loudness; whether it was heard by others; and how it affected the patient. To assess loudness, patients were asked to rate their hip squeaking on a dB scale from the Centers for Disease Control and Prevention (CDC) [21], with patients comparing the level of the loudness to common everyday sounds (Table 1). The dB scale is logarithmic, so a sound at 20 dB is 10 times more intense than a sound at 10 dB. In general, to measure loudness, a sound must be increased by 10 dB to be perceived as twice as loud. We defined the squeaking sound as loud if it was >30 dB. All patients consented to the study and the response rate of patients to the questionnaire was 100%. Implant information and demographic variables including age, sex, diagnosis, and body mass index (BMI) were collected. Anteroposterior pelvis and cross-table lateral hip radiographs were reviewed to determine cup abduction and anteversion angles [22].

**Table 1:** CDC common sources of noise and decibel levels.

Everyday sounds and noises	Average sound level (measured in decibels)
Softest sound that can be heard	0
Normal breathing	10
Ticking watch	20
Soft whisper	30
Refrigerator hum	40
Normal conversation, air conditioner	60
Washing machine, dishwasher	70
City traffic (inside the car)	80-85
Gas-powered lawn mowers and leaf blowers	80-85
Motorcycle	95
Approaching subway train, car horn at 16 feet (5 meters), and sporting events (such as hockey playoffs and football games)	100
The maximum volume level for personal listening devices; a very loud radio, stereo, or television; and loud entertainment venues (such as nightclubs, bars, and rock concerts)	105-110
Shouting or barking in the ear	110
Standing beside or near sirens	120
Firecrackers	140-150

### Statistical analysis

Statistical analysis comparing demographic variables between squeaking and nonsqueaking hips was performed. Subsequently, a statistical analysis comparing loudness, demographic variables, and squeak characteristics was performed for patients who reported squeaking. Student’s *t*-test was used for parametric data, and the chi-squared test was used for categorical data. The Pearson coefficient correlation was used to examine two continuous variables. One-way ANOVAs were used to examine a categorical variable with 3 or more levels and a continuous variable. All statistical analyses were done on IBM SPSS 28 (IBM, Armonk, NY). A *P*-value <.05 was considered to be statistically significant.

## Results

Intermittent squeaking was self-reported to be present in 28% (36 hips) of the cases. Only one hip was reported to have squeaking with all movements of the hip. Of the seven patients with bilateral CoC THAs that reported squeaking, five reported squeaking in only one of their 2 hips, while the other two patients had squeaking in both hips. Four of the patients (4 hips) at a mean of 9.8 years postoperatively (range, 89-149 months) could not recall when their squeaking first started. The mean onset of the squeaking in the remaining 126 THAs was 5.7 years postoperatively (range, 0.5-14 years), with 39% of the cases (14 hips) having their onset of squeaking more than 5 years after their THA.

No significant differences were found in squeaking and nonsqueaking hips in terms of sex (*P* = .65), age (*P* = .36), height (*P* = .58) and follow-up time (*P* = .08) (Table 2). There was a significant difference in BMI between squeaking and nonsqueaking hips (*P* = .01) (Table 2), with an increased incidence of squeaking found in patients with a lower BMI. Squeaking occurred in 24 Trilock BPS/Pinnacle, 11 S-ROM/Pinnacle, and 1 Anthology/R3 THAs. Implant parameters, including acetabular implant size (*P* = .21) (Table 3) and orientation (mean abduction 45° vs 46° and mean anteversion 28° vs 27° in squeaking vs nonsqueaking hips, respectively, *P* = .90), as well as femoral head size (*P* = .19), did not significantly affect the incidence of squeaking (Table 4).

**Table 2:** Demographic data.

Variable	Squeaking (n = 36)	Nonsqueaking (n = 94)	*P*-value
Sex (male: female)	16:20	46:48	.65
Age (y)	52.1	49.8	.36
BMI	24.5	26.5	.01
Height (cm)	171.8	170.7	.58
Months postoperation	116.7	129.1	.08

**Table 3:** Acetabular size.

Acetabular size (mm)	Nonsqueaking (n = 94)	Squeaking (n = 36)
46	1	0
48	5	2
50	10	2
52	38	12
54	16	4
56	9	7
58	11	4
60	2	4
62	2	0
64	0	1

**Table 4:** Distribution of femoral head size.

Cohort	26 mm	28 mm	32 mm	36 mm	*P*-value
Squeaking (n = 36)	1	0	6	29	.19
Nonsqueaking (n = 94)	0	2	15	77	.19

The mean loudness of the squeak was 35 dB (range, 10-70 dB). The squeak was considered loud in 13 of the 36 hips (36%). There was a significant negative correlation between loudness and the years of onset for the squeak, *r* = −0.443 (*P* = .007), with patients who developed squeaking early postoperatively had louder squeaking than those hips with a later onset. Three (8.3%) hips had pain or discomfort associated with the squeak. There was no significant difference in loudness between the squeaking hips with pain or discomfort and those squeaking hips with no pain or discomfort (*P* = .33) (Table 5). In four patients (11%), the squeaking was not associated with pain, but it did cause psychological distress since the sound reminded them of the pain of the surgery, they found the noise quality disturbing, or they were embarrassed by others hearing it.

**Table 5:** Comparison of loudness and pain/discomfort.

Cohort	Mean loudness (dB)	*P*-value
Pain/discomfort (n = 3)	43	.33
No pain/discomfort (n = 33)	34	.33

dB, decibels.

Multiple aggravating positions and activities were associated with the hip squeaking, including hip flexion, twisting, squatting, walking, exercising, and going up or down stairs. The squeaking occurred daily in 7 hips, every other day in 3 hips, weekly in 6 hips, 2 times monthly in 1 hip, monthly in 5 hips, every 2 months in 5 hips, every 3 months in 6 hips, sporadically in 1 hip, and the squeaking stopped in 2 hips. There was no significant difference in the loudness of the squeaking between the patients (23 hips) who avoided movements to prevent squeaking and patients (13 hips) who did not avoid any movements (*P* = .97) (Table 6).

**Table 6:** Comparison of loudness and movement avoidance.

Cohort	Mean loudness (dB)	*P*-value
Avoiding movement (n = 24)	35	.97
Not avoiding movement (n = 12)	35	.97

dB, decibels.

There was a significant difference in loudness between hips that had no change in squeaking frequency and the hips that had increasing squeaking over time (*P* = .04). The 9 hips (25%) in which squeaking became more frequent over time had a louder squeak compared to the 18 hips that did not have a change in squeaking frequency. The mean loudness of the squeaking in hips, in which the squeaking increased, was 44 dB, with 67% of the sound being loud. There was no significant difference between 9 hips that were experiencing less squeaking over time compared to the hips having more squeaking (*P* = .81) or no change in squeaking (*P* = .24).

The loudness of the squeaking varied depending on whether the squeaking could be heard by others and if it was heard occasionally or all the time (Table 7). Patients who reported that others hear the squeaking from their hip sometimes (*P* = .004) or always (*P* < .001) had significantly louder squeaking than those hips in which the squeaking could only be heard by the patient.

**Table 7:** Comparison of loudness between only oneself hearing and others hearing.

Group	Mean loudness (dB)	*P*-value compared to the group
Self (n = 15)	24	Others sometimes: *P* = .004
		Others always: *P* < .001
Others sometimes (n = 14)	38	Self: *P* = .004
		Others always: *P* = .016
Others always (n = 7)	53	Self: *P* < .001
		Others sometimes: *P* = .016

dB, decibels.

## Discussion

This long-term study of CoC THAs found that the mean loudness of squeaking was 35 dB but varied between 10 and 70 dB, with the squeaking being loud in 36% of the hips. Overall, 28% of the hips developed squeaking, starting at a mean of 5.7 years postoperatively. Hips that developed squeaking earlier had louder squeaking than those that started squeaking later. The squeaking loudness progressed in 25% of the cases, and 67% of these cases reported that the sound was loud (>30 dB).

There is considerable variability in the prevalence of squeaking after THA using fourth-generation CoC bearings, likely because of differences in implant design, patient activity, duration of follow-up, and noise assessment methods. Although CoC squeaking can occur early or late postoperatively, 57% of the hips in this study started squeaking at 5 years or later. Similarly, Taniguchi et al. noted that 39% of THAs began squeaking more than 5 years after their initial surgery [8]. Therefore, only studies with longer-term follow-up would be expected to have a similar incidence of squeaking. There are only two other studies that report squeaking with a 4th generation CoC articulation and have a mean follow-up of 10 years or longer [23,24]. With 4th generation CoC bearings, Chatelet et al. reported squeaking in 18% of THAs at 11.1 years of follow-up [24] and Niu et al. found a 12.1% incidence of squeaking at 11 years following cementless THA [23]. This study’s incidence of squeaking of 28% at 10.5 years of follow-up is higher than the other 2 studies. Although these studies involved different implants, the ceramic articulations were identical, and the specific reason(s) for the difference in the incidence of squeaking remains unclear.

To our knowledge, this is the first clinical study to quantify the loudness of CoC squeaking through a dB measurement system. Previous studies [11,13,19,20,24] have classified the noise by its impact on activities of daily living (ADLs) or by the degree to which it was audible to others around the patient. These qualitative descriptions do not accurately indicate the loudness of the squeak and limit the understanding of how various factors affect the severity of squeaking. The CDC dB scale is commonly used in industry and provides a simple scale to quantify everyday sounds with the average sound level they generate. This provided an understandable way for patients in this study to quantify their squeaking. The mean loudness of squeaking in this study was 35 dB, with 36% of the involved hips categorizing the squeaking as loud. Although one can debate how loud the squeak needs to be for it to be problematic, this study found that when the loudness of the squeaking was 24 dB, it could be heard by the patient. When the loudness reached 39 dB, it could be heard by others sometimes, and at 53 dB, it was heard all the time by others.

Several of the findings in this study are in agreement with the previous literature. Similar to studies reporting no or low rates of pain/discomfort in squeaking CoC THAs [5,7,16,24], squeaking was only associated with pain or discomfort in three patients in this study. Patients’ hips that squeaked in this study had lower BMIs, consistent with some studies [7,24,25], but contradictory to others [5,20]. This supports the multifactorial nature of squeaking, as patients with a lower BMI may be more physically active, resulting in increased microseparation, impingement, and/or wear on their CoC articulating surfaces [25,26]. Microseparation can result in the formation of wear stripes along the equatorial surface of the ceramic head, with corresponding accelerated wear along the inner edge of the ceramic liner. This wear pattern then leads to the loss of fluid film lubrication and the generation of audible vibration during various hip motions [25]. Like other studies, our analyses revealed no influence of cup inclination or anteversion on squeaking [24,27].

While our study had several strengths, it also had some limitations. Firstly, the quantification of the loudness of the squeak was based on the CDC dB chart. The most accurate and objective way to measure the dB level of sound is with a sound level meter. Although some patients may have different interpretations of the noises presented in the chart, the CDC dB chart is commonly used by the National Institute for Occupational Safety and Health and industry to quantify sound exposure. Since the chart is not a validated tool in a total hip population, the irrefutable precision of the results can be questioned. Secondly, the technique used to measure acetabular anteversion is not as accurate as a computed tomography scan, which may have resulted in some errors. However, the same technique was used for the hips that squeaked and did not squeak. As well, we did not evaluate the anteversion of the stem. This can be important since the anteversion of the hip is additive, and implant impingement can result in stripe wear and squeaking. However, at the time of surgery, all hips were taken through an extreme range of motion to ensure that there was no impingement of the femoral neck on the edge of the ceramic liner. Finally, these patients were questioned long after their THA and, in some cases, well after their squeaking started. This could have resulted in some inaccuracies in recall regarding when their hips started squeaking. However, patients did not hesitate in responding to the questions since squeaking was discussed with them preoperatively as a significant complication, a dramatic change for the patient, and patients were sensitized to the question of squeaking since it was asked of them at their regular follow-up visit every 2 years.

## Conclusions

At 10.5 years of follow-up, this study found that 28% of hips with a 4th generation CoC articulation experienced squeaking. The loudness of the squeak varied between 10 and 70 dB (mean, 35 dB) and was loud in 36% of the hips. The onset of squeaking occurred at a mean of 5.7 years postoperatively, and the loudness of the squeaking progressed in 25% of the cases. Surgeons should discuss with their patients the risks of developing squeaking long after their CoC THA. This needs to be especially considered for young patients, who are the most common candidates for CoC THAs. Although squeaking is uncommonly associated with pain, the noise can be frequent and disabling if it results in the avoidance of specific activities or discourages social interactions.

## Conflicts of interest

M. Tanzer is a consultant at Stryker, receives educational event honorarium from AO North America, and is also a board member of Canadian Arthroplasty Society. A. Hart receives consulting from Irrimax and is a board member of AO Recon North America; all other authors declare no potential conflicts of interest.

For full disclosure statements refer to https://doi.org/10.1016/j.artd.2023.101203.

## Appendix A Supplementary data

Conflict of Interest Statement for Al Badi

Conflict of Interest Statement for Adam Hart

Conflict of Interest Statement for Zhida Shang

Conflict of Interest Statement for Michael Tanzer

## Appendix

1. Does your hip squeak or make noise (yes/no)?
2. If yes, can you describe the noise/squeak in your own words? ie, squeaking, grinding, popping, clicking
3. Is the noise only heard by you or can it be heard by others - sometimes or always?
4. When did the noise start, and what were you doing at that time?
5. What activities or positions can cause the noise? Does it occur every time you do this?
6. Do you avoid any movements or activities because of this noise?
7. How often does the noise occur?
8. Is the noise continuous or intermittent?
9. Is the noise becoming more frequent, less frequent or is there no change in noise frequency?
10. How would you describe the loudness of the noise?

Normal breathing- 10

Ticking watch - 20

Soft whisper - 30

Refrigerator hum - 40

Normal conversation - 60

Vacuum, traffic - 70

Hair dryer, blender or lawn mower - 90

Motorcycle - 95

Car horn - 110

11. Is the noise associated with any pain or discomfort?
12. Is there anything else you would like to tell me about the noise?
